# Ionizing Radiation-Induced Oxidative Stress Alters miRNA Expression

**DOI:** 10.1371/journal.pone.0006377

**Published:** 2009-07-27

**Authors:** Nicole L. Simone, Benjamin P. Soule, David Ly, Anthony D. Saleh, Jason E. Savage, William DeGraff, John Cook, Curtis C. Harris, David Gius, James B. Mitchell

**Affiliations:** 1 Radiation Oncology Branch, Center for Cancer Research, National Cancer Institute, National Institutes of Health, Bethesda, Maryland, United States of America; 2 Radiation Biology Branch, Center for Cancer Research, National Cancer Institute, National Institutes of Health, Bethesda, Maryland, United States of America; 3 Laboratory of Human Carcinogenesis, Center for Cancer Research, National Cancer Institute, National Institutes of Health, Bethesda, Maryland, United States of America; Roswell Park Cancer Institute, United States of America

## Abstract

**Background:**

MicroRNAs (miRNAs) are small, highly conserved, non-coding RNA that alter protein expression and regulate multiple intracellular processes, including those involved in the response to cellular stress. Alterations in miRNA expression may occur following exposure to several stress-inducing anticancer agents including ionizing radiation, etoposide, and hydrogen peroxide (H_2_O_2_).

**Methodology/Principal Findings:**

Normal human fibroblasts were exposed to radiation, H_2_O_2_, or etoposide at doses determined by clonogenic cell survival curves. Total RNA was extracted and miRNA expression was determined by microarray. Time course and radiation dose responses were determined using RT-PCR for individual miRNA species. Changes in miRNA expression were observed for 17 miRNA species following exposure to radiation, 23 after H_2_O_2_ treatment, and 45 after etoposide treatment. Substantial overlap between the miRNA expression changes between agents was observed suggesting a signature miRNA response to cell stress. Changes in the expression of selected miRNA species varied in response to radiation dose and time. Finally, production of reactive oxygen species (ROS) increased with increasing doses of radiation and pre-treatment with the thiol antioxidant cysteine decreased both ROS production and the miRNA response to radiation.

**Conclusions:**

These results demonstrate a common miRNA expression signature in response to exogenous genotoxic agents including radiation, H_2_O_2_, and etoposide. Additionally, pre-treatment with cysteine prevented radiation-induced alterations in miRNA expression which suggests that miRNAs are responsive to oxidative stress. Taken together, these results imply that miRNAs play a role in cellular defense against exogenous stress and are involved in the generalized cellular response to genotoxic oxidative stress.

## Introduction

Numerous adaptive mechanisms in cells alter gene expression in response to potentially lethal stressors. These mechanisms include the regulation of several fundamental cellular processes including cell cycle progression, gene transcription and translation, as well as epigenetic mechanisms such as DNA methylation or acetylation. miRNAs comprise a highly conserved 18–22 bp RNA family that bind to mRNA and either block transcription or promote mRNA degradation and thus represent a unique mechanism for controlling gene expression. It has been proposed that miRNAs regulate the expression of many mammalian genes. Therefore it is likely that miRNAs regulate several genes involved in the cellular response to potentially lethal stressors.

While it seems likely that there is a mechanistic connection between miRNAs and the response to cellular stress, there have been few studies to date demonstrating this in mammalian cells. In plants it has been shown that changes in miRNA expression appear to be important in tolerance to stresses resulting from changes in environmental conditions including nutrient and water availability, acidity, and temperature [1]. In normal human cells, it has been shown that miRNAs can alter p53 activity [2], and it is now widely reported that miRNA expression is altered in several benign and malignant diseases. Changes in miRNA expression also occur under conditions of hypoxia [3], and are localized to stress response elements in cells subjected to various stressors [4]. Therefore, it seems logical that miRNAs may play a role in the preprogrammed intracellular response to exogenous cytotoxic agents that induce oxidative stress.

Ionizing radiation is an important modality used in the treatment of malignancy and is one example of an agent that induces oxidative genotoxic stress. Ionizing radiation causes severe cellular damage and stress both directly, by energetic disruption of DNA integrity, and indirectly, as a result of the formation of intracellular free radicals. A small number of studies have shown an effect of radiation on miRNA expression patterns both *in vitro* [5], [6] and *in vivo* [7], [8]. Two studies have shown that radiation sensitivity can be altered by upregulating the expression of a single miRNA species [5], [9]. However, the mechanism underlying the effect of radiation on specific miRNA expression remains to be elucidated.

In this study, we show that alterations in miRNA expression due to ionizing radiation are also produced in response to other agents that induce either DNA damage or oxidative stress. We also show that the miRNA response to radiation can be abrogated by the addition of the free radical scavenger cysteine. These data suggest that both DNA damage and free radical formation provoke what appears to be a common miRNA expression signature of exogenous genotoxic stress. It is likely that the miRNA response to stress utilizes mechanisms similar to those that govern other cellular stress responses, however further studies are needed. Finally, the directed modulation of miRNA expression using miRNA-specific agonists and antagonists may be a useful clinical tool to alter the response of tumors and normal tissue to the effects of radiation.

## Materials and Methods

### Cell Culture and Treatments

Normal human fibroblasts (AG01522) (Coriell Institute for Medical Research, Camden, NJ) were maintained in F12 media supplemented with 20% fetal bovine serum, 100 units/mL penicillin, and 100 µg/mL streptomycin at 37°C and 5% CO_2_ and used at an early passage. Cells were plated into 100-mm dishes and incubated for 2 days then treated while in logarithmic growth with either ionizing radiation, etoposide (100 µM) for 1 hour, or H_2_O_2_ (25 µM) for 1 hour. The doses of etoposide and H_2_O_2_ used were determined by clonogenic survival assay to produce one log cell kill. To evaluate the effect of a radiation modifier on miRNA expression following irradiation, cells were treated with the free radical scavenging agent, cysteine (1 µM) and catalase (100 µM), 1 hour prior to and during the irradiation exposure.

### Clonogenic Cell Survival Assays

The effect of ionizing radiation, etoposide, or H_2_O_2_ on cell survival was determined by clonogenic assay. To determine the toxicity of etoposide, fibroblasts were maintained in culture and then seeded at densities of 5×10^4^ cells per well in a 6-well plate and allowed to grow for three days at 37°C and 5% CO_2_. Etoposide was added to the media at doses of 1, 5, 10, and 25 ug/ml. After 1 hour, 24 hours, and 48 hours of exposure to etoposide, the cells were trypsinized, diluted, counted, and seeded into 6-well tissue culture plates at densities of 250 or 1000 cells per well. Colonies were allowed to form from surviving cells for 10 days, after which they were fixed, stained, and counted. A dose response to ionizing radiation and H_2_O_2_ was determined using a similar method.

### Irradiation of Cells in Culture

Normal human fibroblasts were irradiated using an Eldorado 8 ^60^Co teletherapy unit (Theratronics International Ltd., Kanata, Ontario, Canada) at a dose rate of approximately 200 cGy/min. Cells were irradiated to the total dose called for in the experimental design for each experiment. Decay corrections were performed monthly, and full electronic equilibrium was ensured for all irradiation. The radiation doses used in these experiments were chosen to cover a wide range which included low doses (0.25, 0.5, 0.75, and 1 Gy) to high doses (3, 5, and 10 Gy).

### Detection of Reactive Oxygen Species (ROS)

ROS were measured in a 96-well plate assay using the fluorescent probe dichlorofluorescein (DCF) [10]. Fibroblasts (5×10^5^/ml) were treated with cysteine and catalase as above then incubated with DCF diacetate (20 µM) in cell culture medium for 15 min at 4°C with rotation. Cells were then washed in cold PBS and seeded at 200,000 per well in a black opaque 96-well microplate. Measurement of DCF fluorescence was initiated within 1 min after irradiation and was monitored for 15 min using a GENios fluorescent plate reader (ReTirSoft) set at an excitation wavelength of 492 nm and emission wavelength of 535 nm. Fluorescence was expressed as relative fluorescent units and area under the curve (AUC) for the plot of fluorescent units over time.

### Collection and Preparation of miRNA

For most experiments, cells were collected 1 hour after treatment with either radiation, etoposide, or H_2_O_2_. For studies of the response of miRNAs to radiation over time, cells were collected at various times after radiation, as called for in the experimental design. The cells were rinsed in PBS then directly lysed and collected using Trizol reagent (Invitrogen, Carlsbad, CA), from which total RNA was isolated using a standard phenol-chloroform extraction method. Total RNA was quantified using a NanoDrop spectrophotometer (Thermo Fisher Scientific, Wilmington, Delaware).

### miRNA microarray and real-time PCR

miRNA microarray analysis was performed by LC Sciences (Houston, Texas) on samples that included an untreated control, and samples treated with radiation, etoposide or H_2_O_2_ as previously described [11]. Microarrays utilized µParaflo™ microfluidic chip technology and optimized RNA hybridization probes with a detection limit of <100 attomole that was cross referenced to the Sanger miRNA database (Release 9.0, microrna.sanger.ac.uk/) after normalization. For the purposes of this assay, the expression of a miRNA species was determined by calculating the ratio (log transformed) of the detected control and sample signals, and calculating the p-values of the t-test. Differentially detected signals were generally accepted as true when the ratios of the p-value was <0.01 and were then selected for cluster analysis. The data from the microarray was collected and analyzed in accordance to the MIAME guidelines. To confirm the microarray results, RT-PCR was performed on several samples. Using the TaqMan MicroRNA Assay Kit (Applied Biosystems, Foster City, CA), which utilizes miRNA-specific primers, we reverse-transcribed 10 µg of each RNA sample according to the manufacturer's instructions. The resulting cDNA was semiquantitatively amplified in 45 cycles on an ABI 7500 Real-Time PCR System, using TaqMan Universal PCR Master Mix No Amp Erase UNG and Taqman MicroRNA Assays for let-7a, let-7b and 18S (Applied Biosystems, Foster City, CA).

### Analysis

Reverse transcriptase reactions were performed after standard curves were derived for each miRNA of interest. Statistical analysis was performed using Student's t-test, and p<0.05 was considered statistically significant. Each experiment was performed at least 3 times and individual samples were run in triplicate.

## Results

### miRNA Microarray

**Figure 1:**
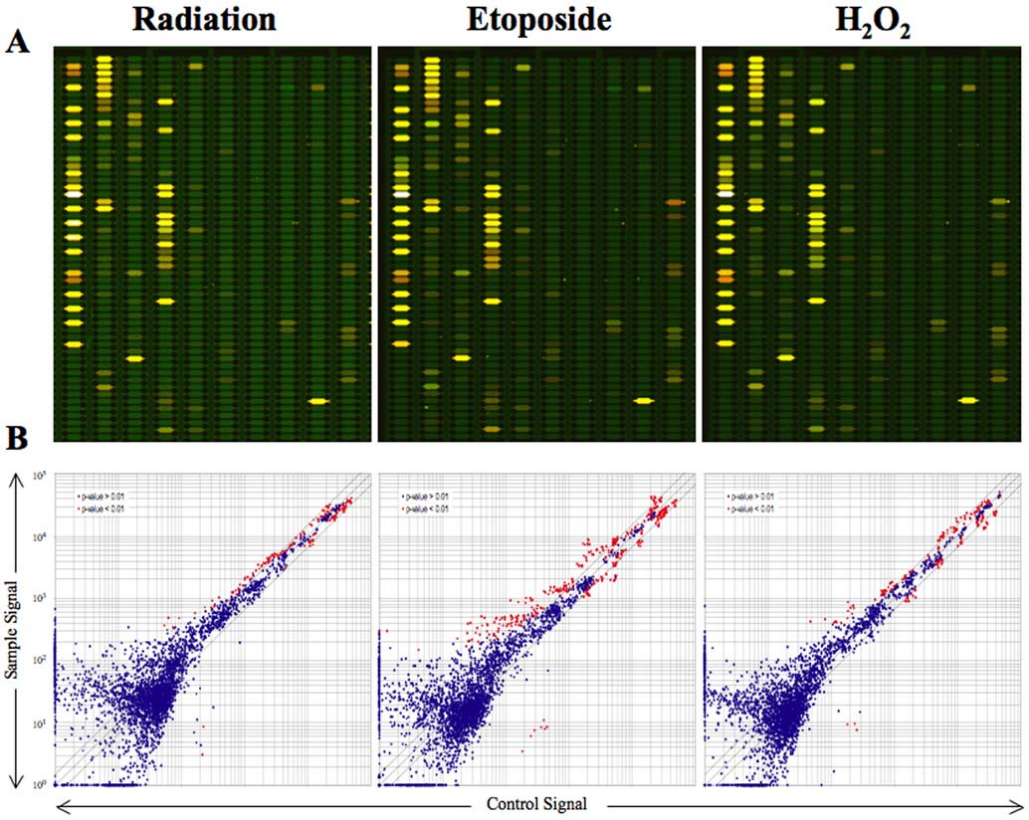
miRNA Micorarray changes following exposure to radiation, H_2_O_2_, and etoposide. 1522 cells were collected one hour after exposure to 10Gy of radiation, etoposide, or H_2_O_2_. The microarray (A) was performed using RNA isolated from treated cells and compared to RNA from untreated controls. (B) Scatter plot of miRNA expression in control versus radiation, etoposide, or H_2_O_2_ samples.

**Figure 2:**
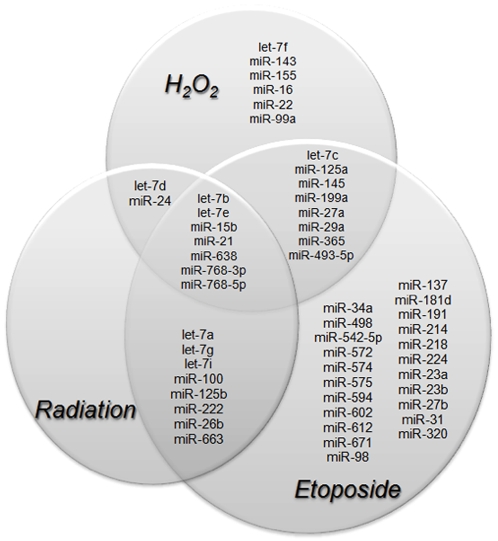
Venn Diagram showing miRNA species altered by stressors. 1522 cells were collected one hour after exposure to 10Gy of radiation, etoposide, or H_2_O_2_. miRNAs with expression that is significantly different from control as determined by microarray are shown illustrating the overlapping signature of exogenous genotoxic stresses.

To determine if miRNA expression may be altered in response to exogenous agents that, at least in part, induce intracellular oxidative stress, 1522 primary human fibroblasts were exposed to doses of ionizing radiation, H_2_O_2_, or etoposide that had been previously determined to generate one log of cell kill by the clonogenic assay. After exposure, cells were analyzed using a microarray assay to probe for all known human miRNA species identified in the Sanger miRBase (Fig. 1). This analysis revealed that irradiation alters the expression of 17 miRNA species using a strict cutoff of p<0.01 for significance. A more extensive analysis also determined that all 17 miRNA species altered by radiation were also altered by either H_2_O_2_ or etoposide (Table 1). Several species were altered only by etoposide or H_2_O_2_ (Fig. 2).

**Table 1:** Species with Altered miRNA Expression.

	Radiation	H_2_O_2_	Etoposide
let-7d	+0.39	+0.74	n/s
miR-24	−0.38	−0.52	n/s
let-7a	−0.40	n/s	−0.48
let-7g	+0.85	n/s	+1.27
let-7i	+0.79	n/s	+0.81
miR-100	−0.5	n/s	−0.79
miR-125b	−0.33	n/s	−0.45
miR-222	−0.38	n/s	−0.29
miR-26b	+0.98	n/s	+1.09
miR-663	+0.79	n/s	+2.00
let-7b	−0.32	+0.37	−0.74
let-7e	+0.43	+0.82	+0.43
miR-15b	+0.39	+0.74	−0.40
miR-21	+0.51	−1.01	+0.81
miR-638	−0.48	−0.76	+0.46
miR-768-3p	+0.82	+0.91	+0.91
miR-768-5p	+0.87	+0.89	+0.79

miRNA microarrays were run using 1522 cells treated with either radiation, H_2_O_2_ or etoposide. miRNA species that were significantly changed (p<0.01) from control are shown in this table. Expression of 17 miRNA species was significantly altered by radiation. Of these, all were also altered by treatment with H_2_O_2_, etoposide, or both. The reported values were calculated as the log_2_(sample/control) for assessment of differential direction as well as magnitude with differentially expressed species with p<0.01 (n/s denotes miRNA species that were not significantly changed).

Expression levels in 10 of the 17 miRNA species increased after exposure to radiation, whereas expression of 7 species decreased. In all cases, the direction of the change in miRNA expression in radiation-treated cells correlated with the change observed in cells treated with either H_2_O_2_ or etoposide (Table 1). For 7 miRNA species, expression was altered by all three treatments and in 3 of those 7 cases (let-7b, let-7e, and miR-15b) the direction of the change in expression was the same for all three treatments. In 4 of the 7 cases (miR-21, miR-638, miR-768-3p and miR-768-5p), the direction of the change in expression was not consistent across all three treatments, however in all 4 cases the direction of the change induced by radiation was also induced by either etoposide or H_2_O_2_. These results suggest that there may be a common miRNA expression signature that is altered by agents that induce cytotoxicity and also, at least in part, induce oxidative stress.

### miRNA Real-Time PCR validation of miRNA Stress Response

**Figure 3:**
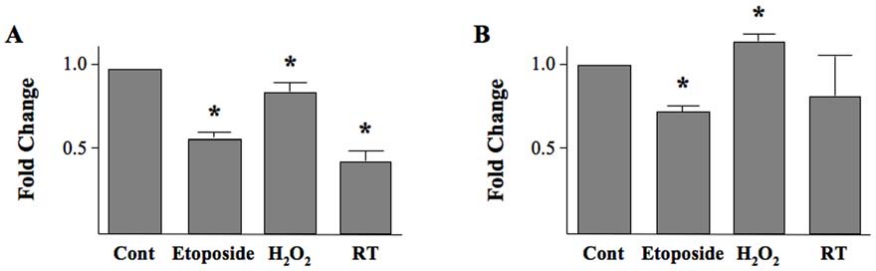
Response of miRNAs to genotoxic stress. 1522 cells were collected one hour after exposure to 10Gy of radiation, etoposide, or H_2_O_2_ and evaluated by RT-PCR for expression of (A) let-7a and (B) let-7b. A decrease in let-7a expression is noted after treatment with all three agents. A decrease in let-7b expression is noted for radiation and etoposide while an increase in expression is noted after H_2_O_2_ treatment. All results represent the average of three separate experiments all done in triplicate. Error bars represent one standard deviation about the arithmetic mean and * denotes p<0.05.

To confirm the microarray data results, the changes in miRNA expression for let-7a and let-7b were validated by RT-PCR. These miRNAs were chosen since they are presumed, by sequence homology analysis, to target the *ras* pathway, which is known to be affected by radiation [12], [13]. Expression of both let-7a and let-7b decreased significantly after treatment with radiation and etoposide. A decrease in let-7a expression and an increase in expression of let-7b was observed in cells treated with H_2_O_2_ (Fig. 3).

### Changes in miRNA expression related to radiation dose

**Figure 4:**
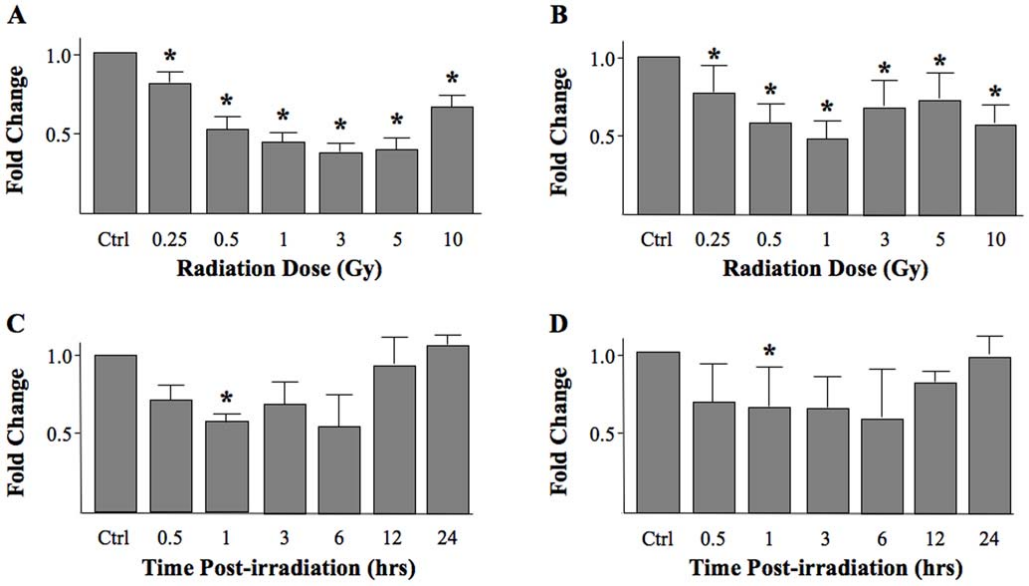
Response of miRNAs to radiation dose and time after radiation. 1522 cells were irradiated to doses ranging from 0.25Gy to 10Gy. Samples were collected one hour after irradiation and evaluated by RT-PCR for expression of (A) let-7a and (B) let-7b. A dose-dependent, linear decrease in expression from 0.25Gy to 1Gy was noted with no further response seen at higher doses. In addition, RT-PCR was performed on samples collected at several time points ranging from 30 min to 48 hrs after irradiation (10Gy). A decrease in (C) let-7a and (D) let-7b expression was noted at 30 min with a return to baseline expression by 24 hrs after irradiation. All results represent the average of three separate experiments all done in triplicate. Error bars represent one standard deviation about the arithmetic mean and * denotes p<0.05.

It is well established that changes in gene expression can vary significantly following exposure to subclinical, clinical, and super-clinical radiation doses. Thus, we determined the dose response effect of ionizing radiation on the changes in miRNA expression levels. Cells were exposed to radiation at doses ranging from 0.25 to 10 Gy revealing a dose-dependent, linear decrease in miRNA expression from 0.25 to 1 Gy one hour after irradiation (Fig. 4). No further dose-dependent decrease was noted in the higher dose ranges. The results of these experiments suggest that miRNA expression does indeed change with radiation dose and that 1 Gy may produce the maximum alteration in let-7a and let-7b.

### The timing kinetics of miRNA following exposure to irradiation

Alterations in gene expression following radiation exposure appear to change as a function of time and these changes have been proposed to be a potential marker that might better guide the delivery of therapeutic irradiation. As such, the pattern of miRNA changes with respect to time was evaluated. RT-PCR was performed using cells collected at several time points after radiation exposure ranging from 15 minutes to 48 hours. These experiments demonstrated variability in miRNA expression over time (Fig. 4). miRNA expression decreased thirty minutes following irradiation and remained reduced through the 6-hour time point. Twelve hours after radiation exposure, miRNA expression began to increase and returned to baseline at 24 hours. This pattern was observed for both let-7a and let-7b.

### Cysteine prevents the changes in ROS production and let-7a and let-7b expression

**Figure 5:**
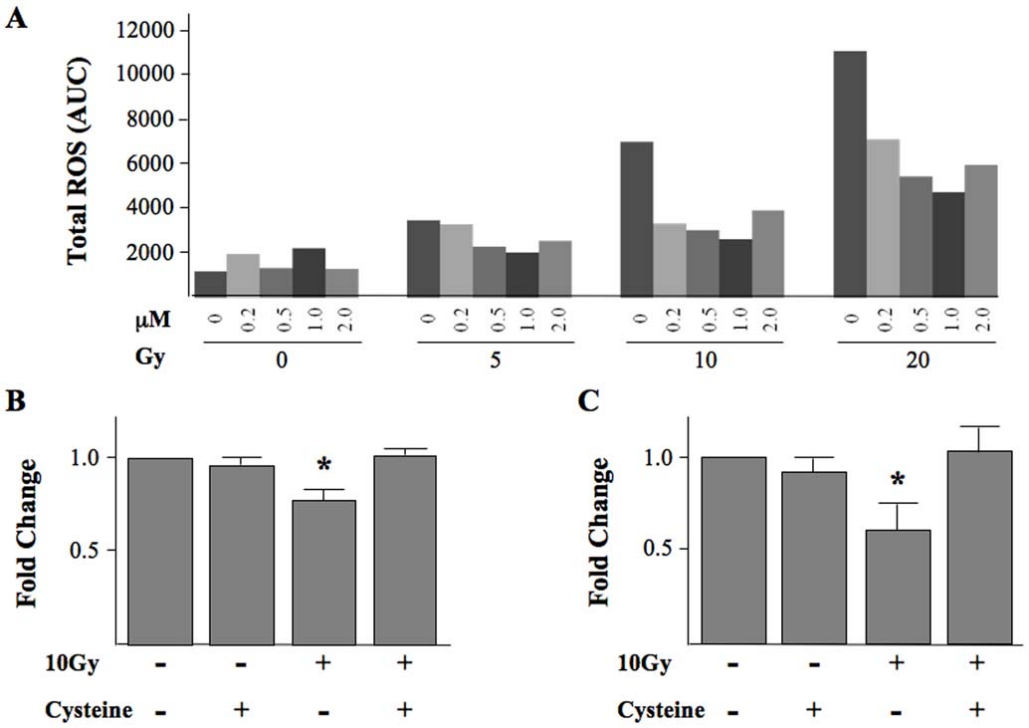
The Effect of the Free Radical Scavenger Cysteine on miRNA expression. (A) 1522 cells were treated with cysteine (0.2, 0.5, 1 or 2 µM) for one hour, then DCF (dichhlorofluroisceine) for 15 min, then irradiated to 5, 10 or 20Gy. ROS expression, measured for 15 min after irradiation, increased with increasing radiation dose and decreased with increasing doses of cysteine. ROS expression is reported as area under the curve (AUC) for plot of luminescence intensity over time. Subsequently, 1522 cells were collected one hour after treatment with cysteine (1 µM) alone, radiation (10Gy) alone, or cysteine added 1 hour prior to irradiation. Cysteine alone did not alter miRNA expression, however pre-treatment with cysteine prevented a radiation-induced decrease in (B) let-7b and (C) let-7a expression. All results represent the average of three separate experiments all done in triplicate. Error bars represent one standard deviation about the arithmetic mean and * denotes p<0.05.

It is well established that ionizing radiation, as well as other exogenous genotoxic agents, induce intracellular signaling pathways and changes in gene expression via the generation of reactive oxygen species (ROS). Cysteine abrogates the effects of radiation by scavenging free radicals. We determined whether cysteine itself altered miRNA expression or if it could prevent the alterations in miRNA expression caused by radiation. The production of ROS by fibroblasts was measured after irradiation to 2, 5, or 10 Gy. Measurement of ROS production was initiated within 1 min after irradiation but had already reached a plateau level that was dependent on the radiation dose. Fig. 5A shows that total ROS production, expressed as area under the curve, immediately following irradiation was significantly increased as compared with nonirradiated cells, and that pretreatment with cysteine substantially reduced radiation-induced ROS production in a dose-dependent manner. A small increase in ROS production after treatment with 2 µM cysteine compared to 1 µM cysteine represents auto-oxidation caused by cysteine hence 1 µM cysteine was chosen for subsequent experiments.

Pre-treatment of cells with cysteine prevented the radiation-induced decrease in let-7a and let-7b expression (Fig. 5) and no change in expression was observed in cells treated with cysteine alone. These results suggest that oxidative stress may play a role in the alteration of miRNA expression due to radiation.

## Discussion

In this study, we show that alterations in miRNA expression due to ionizing radiation are also produced in response to other agents that induce either DNA damage or oxidative stress. Furthermore, there appears to be substantial overlap between the specific changes in miRNA expression. The miRNA species that are changed by radiation are also altered after treatment with H_2_O_2_, which imposes a free radical stress, or with etoposide, which generates double strand breaks mimicking the direct DNA damage caused by radiation. Therefore, it seems reasonable to suggest a potentially common miRNA signatures in response to these genotoxic agents.

Two miRNA species were altered by both radiation and H_2_O_2_ and eight species were altered by both radiation and etoposide. In each instance, the direction of the change in expression following radiation exposure correlated with the direction of the change in expression resulting from either H_2_O_2_ or etoposide. The expression of seven miRNA species was changed by all three genotoxic agents and, once again, the direction of the change resulting from ionizing radiation corresponded with the direction of the change caused by either etoposide or H_2_O_2_. Furthermore, the magnitude of the change in expression of selected miRNA species correlated with both the radiation dose and with time after radiation exposure. We also show that the response to radiation can be abrogated by the addition of the free radical scavenger cysteine. Taken together, these data suggest that miRNA expression is responsive to cell stress resulting from both DNA damage and free radical formation provoking what appears to be a common expression signature of exogenous genotoxic stress.

Since miRNAs control gene expression at the post-transcriptional level, they enable the cell to alter expression of a gene product very rapidly compared to mechanisms that alter transcriptional activity or protein degradation. The association with radiation dose also suggests that this rapid response can be modulated to respond to the severity of the insult. This mechanism may allow a cell to respond with a preprogrammed response to extracellular signals and stressors.

It has previously been shown that several miRNA species are associated with DNA repair processes. For example, miR-106b has been shown to regulate the p21 checkpoint and can, therefore, either promote cell cycle progression or cause cells to accumulate in the G1 phase [14]. Similarly, miR-17 and miR-20a also can affect G1 checkpoint regulation by means of the transcription factor E2F1 [15], and miR-34 has been shown to regulate p53 [2]. Taken together with our observations, this suggests that miRNAs may play an important role in DNA repair after radiation-induced damage. This is further supported by the fact that several of the miRNA species that are altered by radiation have potential targets that are involved in DNA repair including cyclin-dependent kinase 5 (miR-15b), topoisomerase I, histone 2AX (H2AX), and cyclin-dependent kinase inhibitor I (miR-24), and phosphatase and tensin homolog (PTEN) (miR-26b) [16].

Since miRNAs are responding to radiation, oxidative stress, and direct DNA damage, it is logical to suggest that miRNA expression is altered by, and may regulate, pathways involved in cellular stress. This was demonstrated in one study which identified a set of hypoxia-regulated miRNA species that were induced by hypoxia in breast and other cancer cell lines causing hypoxia-inducible factor (HIF) to interact with miRNA promoters [3]. miRNA expression also appears to be responsive to pro-inflammatory signals [17], changes in osmolarity [18], cardiomyocyte stress associated with heart failure [19], and several species have been shown to localize to stress response elements in cells subjected to various stressors [4]. In a recent study, [5] the authors increased or decreased miR-521 expression and found that the levels of the DNA repair protein Cockayne syndrome protein (CSA) and the antioxidant manganese superoxide dismutase were altered after irradiation in prostate cancer cell lines. Furthermore, two studies have demonstrated altered susceptibility to radiation in cells overexpressing a single miRNA species [5], [9].

Our data suggest that both changes in intracellular oxidation/reduction status and damage to DNA may alter miRNA transcription, however the mechanism underlying this remains unclear. Since all three agents that were tested have previously been shown to induce genetoxic and oxidative stress, this suggests to us that the miRNA response utilizes mechanisms involved in the response to other cellular stresses including both changes in intracellular oxidation/reduction status and damage to DNA. For example, many mechanisms exist that alter transcriptional activity in response to various stressors. These include well-described redox-responsive alterations in activity of several transcription factors including NF-κB [20], AP-1 [21], and OxyR [22]. Many proteins are also directly responsive to DNA damage, including those responsible for DNA repair. This response often appears to act through post-translational modifications such as phosphorylation which activates the DNA repair protein H2AX [23] and the DNA helicase RECQ1 [24]. Further studies will be required to confirm whether these mechanisms also control transcription factors or other elements that control miRNA expression changes in response to cell stress.

miRNAs are novel, highly conserved modifiers of gene expression that are responsive to various stressors including free radical stress, DNA damage, and ionizing radiation. It is clear that they represent an important mechanism by which cells can rapidly alter gene expression to respond to potentially lethal stress however the mechanisms underlying this response remain unproven. As such, the directed modulation of miRNA expression may be a useful clinical tool to alter the response of tumors and normal tissue to the effects of radiation. Agonists and antagonists of miRNAs are being developed that may have a role in altering the response of both normal and malignant tissue to the effects of radiation. Further studies investigating specific miRNA responses, elaborating the mechanisms underlying these responses, and validating these findings *in vivo* are under way.
